# Apparent stabilizing selection explains micro- and macroevolution of *Drosophila* wings

**DOI:** 10.1038/s41559-026-03121-2

**Published:** 2026-07-10

**Authors:** Anneli Brändén, Stephen P. De Lisle

**Affiliations:** Department of Environmental and Life Sciences, Karlstad University, Karlstad, Sweden

**Keywords:** Evolution, Evolutionary ecology

## Abstract

Evolution over deep time is often slow relative to neutral or directional expectations. A striking example is provided by *Drosophila* wing shape, which shows substantial genetic variance yet comparatively little macroevolutionary change. Here, using a large-scale fitness assay, we find pervasive stabilizing selection across dimensions of *Drosophila melanogaster* wing shape that is proportional to standing variation, driven by variance in male fitness and probably apparent selection generated by pleiotropy. Parameterizing a macroevolutionary Ornstein–Uhlenbeck model using our selection estimate recapitulates observed among-species variance under reasonable effective population sizes, suggesting a role for apparent stabilizing selection in constraining wing shape evolution over deep time.

## Abstract (web summary)

Analysis of fitness and wing shape in laboratory-bred *Drosophila* reveals signatures of apparent stabilizing selection against extreme wing shapes, consistent with deep-time divergence in wing shape across the Drosophilidae clade.

## Main

Stabilizing selection is a fundamental process in any explanation of evolutionary change over long timescales. In most lineages, ample genetic variance means that expectations for evolution under neutral genetic drift or directional selection result in wildly high predictions for divergence over even moderate timescales^1–3^. Without discounting a supporting role for developmental and genetic constraints, no explanation for macroevolutionary stasis is complete without invoking a leading role for some form of stabilizing selection^3–5^. Surprisingly, there have been remarkably few^6,7^ empirical attempts to directly assess the role of stabilizing selection in generating patterns of stasis.

**Extended Data Fig. 1:**
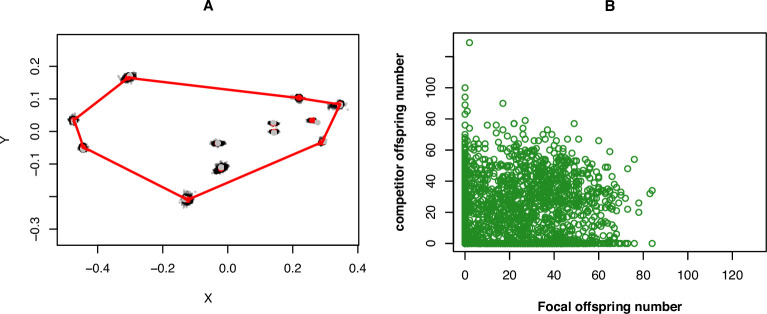
Variation in wing shape and fitness in *D. melanogaster*. Panel **A** shows wing shape variation represented by 12 landmarks demarking major vein intersections, here scaled and rotated in a Procrustes analysis (N = 1994). Panel **B** shows focal and competitor offspring number from a competitive mating assay; this is a binomial measure of competitive reproductive success (N = 2130).

Here we report the results of a large-scale assay (phenotypic scores on 91,632 offspring from 2,110 parents) of individual fitness and wing phenotype in a laboratory-adapted lineage of fruit fly, *Drosophila melanogaster*. Fruit fly wings (Extended Data Fig. 1) exemplify how the perennial puzzle of macroevolutionary stasis has itself evolved when confronted with more (and more sophisticated) datasets. Wing shape has remained remarkably unchanged across the Drosophilidae^4^ and beyond^8,9^, despite substantial genetic variance across all dimensions of measured wing shape^10^. Intriguingly, within-species patterns of mutational and standing genetic variance are conserved in the form of among-species evolutionary rate; wing shape combinations with high mutational input exhibit high standing genetic variance within species and high macroevolutionary rate among species^11^ (but see ref. ^12^). These studies highlight some role for mutational constraints^11^ and developmental bias^8,9^ in linking micro- and macroevolution^13^, but importantly the same studies^11^ also note that some form of stabilizing selection on wing shape must operate to generate the patterns observed. Across a range of taxa, a related finding that within-species variance is a strong predictor of macroevolution can only be fully resolved by invoking stabilizing selection^3^.

**Fig. 1:**
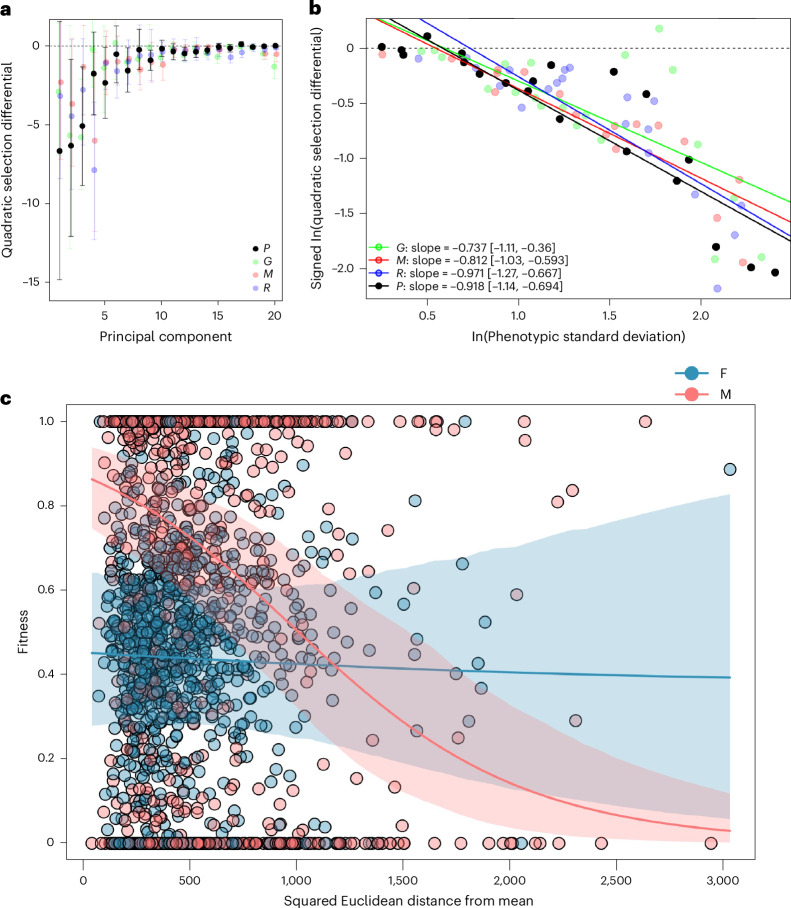
Pervasive stabilizing selection on wing shape is proportional to phenotypic variance. **a**, Nonlinear selection differentials for raw phenotypic scores on the principal components of the phenotypic (*P*), genotypic (*G*), mutational (*M*) and macroevolutionary (*R*) covariance matrices. **b**, Signed-log-log regressions of quadratic selection differentials against the phenotypic standard deviation of the principal component, with regression slopes and 95% CIs shown in the inset. **c**, Sex-specific selection against total Euclidean distance from the mean (calculated as the sum of squared scores on the principal components of *P*). *n* = 1,806 flies (Methods). F, female; M, male.

**Extended Data Fig. 2:**
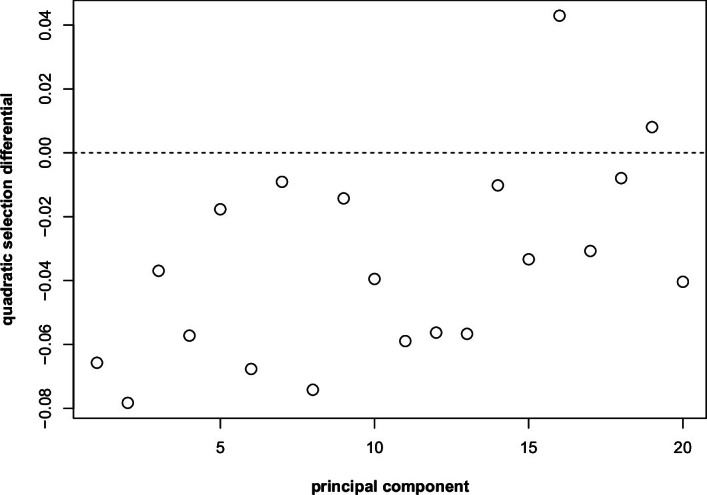
Results are robust to variance standardization. Point estimates of nonlinear selection differentials for phenotypic scores on the principal components of the **P** matrix, where the scores for each component have been variance standardized prior to estimation of the selection differentials.

**Extended Data Fig. 3:**
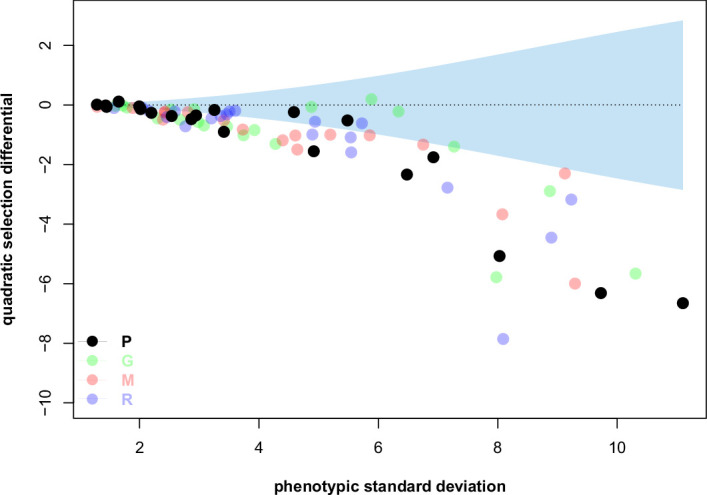
Selection estimates plotted against simulated null expectation. Shaded band shows 95% prediction limits from simulations under null nonlinear selection, across the range of phenotypic variation observed. For details, see Methods. Empirical estimates of selection are as described in Fig. 1.

**Extended Data Fig. 4:**
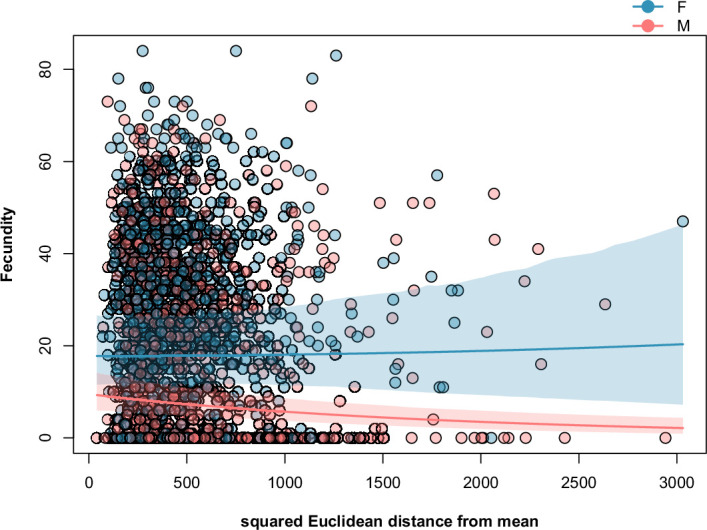
Sex-specific selection against phenotypic extremes, assuming raw fecundity as a fitness measure. Solid lines show predicted values and bands show 95% prediction limits from a Poisson generalized mixed model, with otherwise-identical structure that that fitted for Fig. 1b.

**Extended Data Fig. 5:**
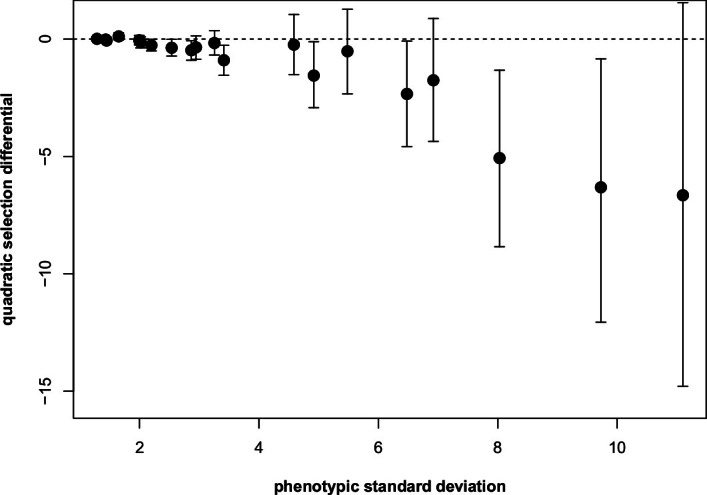
Pervasive stabilizing selection remains when accounting for centroid size. Unstandardized quadratic selection differentials for phenotypic scores on principal components of **P**, plotted against the corresponding phenotypic standard deviation. From GLMMs that include linear and quadratic terms for log centroid size, otherwise fitted as in Fig. 1.

We find pervasive stabilizing selection on multivariate wing shape in *D. melanogaster*. Assessing the same landmark coordinates rotated into the same shape space as past work^8,9,11^, as well as competitive lifetime reproductive success in the same individual flies, we find that estimates of quadratic selection differentials are nearly universally negative across dimensions of wing shape and related strongly to the amount of phenotypic variance (Fig. 1a,b; controlling for sex and block). Although the pattern is strongest when selection is estimated on phenotypic scores on the principal components of the phenotypic covariance matrix *P*, we find a similar pattern when selection is inferred on phenotypic scores on principal components of an existing^11^ estimate of the genetic covariance matrix *G*, the mutational covariance matrix *M* and the macroevolutionary rate matrix *R*; for all four sets of phenotypic scores, signed-log-log regression of estimated selection differentials on the phenotypic standard deviation yields slopes that are significantly negative (all *P* < 0.001) with confidence intervals overlapping −1 and each other estimate (Fig. 1b). These results (which are robust to variance standardization of the differentials, Extended Data Fig. 2; and which also cannot be explained by random sampling across traits differing in variance, Extended Data Fig. 3) indicate that the amount of stabilizing selection is proportional to the amount of variation in any given combination of wing shape traits, consistent with a model of apparent stabilizing selection against total multivariate distance from the mean wing shape (as proposed by ref. ^12^). To assess this conjecture directly, we fit models of selection on total squared Euclidean distance (calculated as the sum of squared principal component (PC) scores for each individual). We found significant selection against multivariate distance (model without sex interaction; generalized linear mixed model (GLMM) coefficient = −0.001142, CI = [−0.001684, −0.000591], *P*_mcmc_ < 0.001) that is sex specific; selection is an order of magnitude stronger in males than in females (model with sex interaction; GLMM interaction coefficient = −0.001844, CI = [−0.002941, −0.000748], *P*_mcmc_ < 0.001; Fig. 1b). We obtain the same conclusions in an analysis of fecundity (Extended Data Fig. 4) and when controlling for centroid size (Extended Data Fig. 5). Together, these results indicate a pattern of stabilizing selection against extreme wing shape, driven primarily by variance in male fitness.

**Extended Data Fig. 6:**
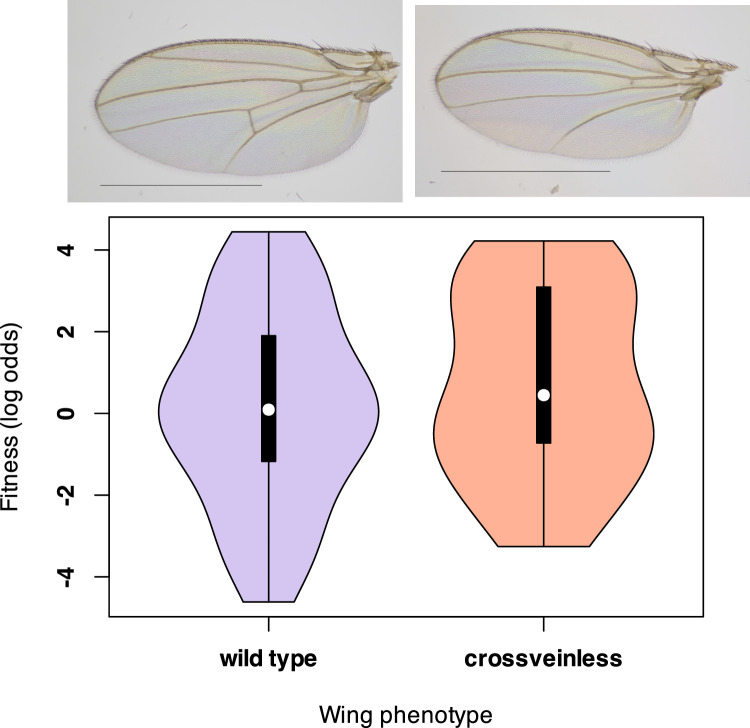
No selection against missing crossveins. Shown are the log odds of fitness for flies with wild type and crossveinless wings. A binomial generalized mixed effects models revealed no evidence of selection against the cross veinless phenotype. Violin plots show kernal density (colored area), median (white dot), interquartile range (black box), and range (whiskers). Wildtype N = 1940, crossveinless N = 29. Scale bars indicate 1 mm.

We propose that the stabilizing selection we have observed is probably apparent and generated by pleiotropic effects of segregating variants on other traits directly affecting fitness^14–16^. Three lines of evidence support this interpretation. First, we have measured selection on wings in a benign laboratory environment to which the population is well adapted (>500 generations), an environment in which strong direct selection on wing shape is not expected. Although males can use wings to sing and display, interference colour display patterns are largely orthogonal to wing shape^17^, and there is unlikely to be selection on flight performance in standard fly vials in which fitness was assayed. Second, we find that stabilizing selection is proportional to standing variance, a pattern consistent with apparent selection via pleiotropy; under such a model of apparent selection, stabilizing selection is predicted to be highest on trait combinations with high variance^14^, as segregating variants affecting these combinations by definition are highly pleiotropic and are most likely to have large pleiotropic effects on other traits that directly affect fitness. Although this concept has been developed^14^ in the context of apparent stabilizing selection on phenotypic scores on *g*_max_, the same reasoning extends beyond the first principal component of variation. Indeed, ref. ^12^ developed such a simulation model of apparent selection via pleiotropy on total Euclidean distance from the mean to explain stasis in fly wing shape; our estimate of selection provides direct empirical support for their model. Third, we find no detectable selection on major deviations in the form of wing vein structure itself: in an analysis of 29 flies (12 female, 17 male) exhibiting cross-veinless phenotypes, we find no reduction in fitness compared with wild-type (*n* = 1,940) vein structure (effect of veinlessness = 0.5278, CI = [−0.9609 2.0344], *P*_mcmc_ = 0.49; Extended Data Fig. 6). Crossveinlessness can be an outcome of environmental effects^18^ or major effect mutation^19^ and, in either case, if wing structure itself was a direct target of selection, we would expect a fitness effect of this phenotype. Thus, lack of selection on gross wing vein structure, but detectable stabilizing selection on quantitative wing shape variation, is most consistent with a model of apparent stabilizing selection on shape via pleiotropy.

**Fig. 2:**
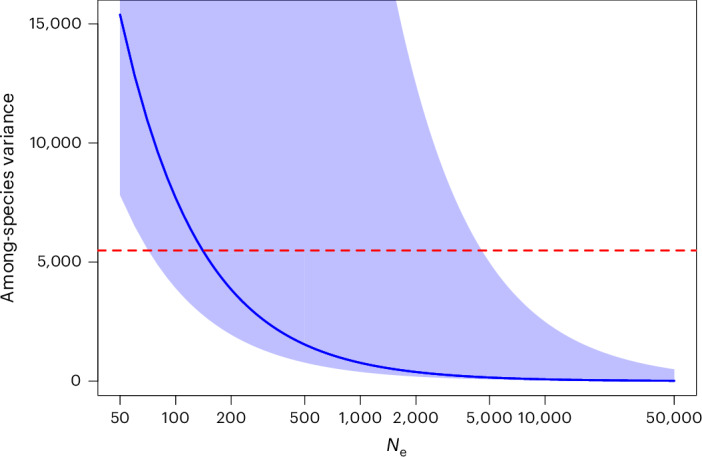
A macroevolutionary model of genetic drift plus stabilizing selection can explain low levels of observed divergence across the clade. The stationary variance (predicted among-species variance at equilibrium) from an OU model parameterized with the estimate of multivariate stabilizing selection (pooled by sex) as a function of *N*_e_. Shaded area shows the 95% CI of the estimate of selection; dashed red line shows the observed phenotypic variance among species.

**Extended Data Fig. 7:**
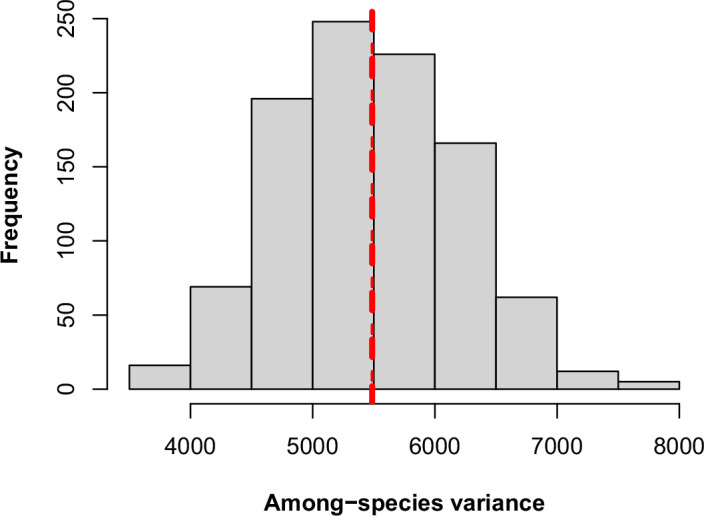
Correspondence between theoretical stationary OU variance and simulated OU evolution on the Drosophilid phylogeny. Red line shows observed among-species phenotypic variance. Histogram shows frequency distribution of simulated (1000x) OU evolution on the rescaled Drosophilid phylogeny assuming calculated from the intersection of the two lines in Fig. 2 (see Methods).

Such an inference of apparent stabilizing selection in an adapted population represents a background force expected to constrain divergence. We can then assess directly whether our estimate of total stabilizing selection (ref. ^20^ estimate of *γ* = −0.00017, 95% CI = [−0.00033, −0.0000053], not variance standardized), when combined with inference of standing genetic variance^11^, is generally consistent with patterns of deep-time divergence in wing shape across the Drosophilidae. To do this we explore the simplest model of divergence under stabilizing selection, an Ornstein–Uhlenbeck (OU) process^21^, where lineages diverge under genetic drift proportional to $\frac{G}{{N}_{\rm{e}}}$, and are pulled back towards the optimum by *α*. Here *α* is the curvature of the adaptive landscape, which can be defined^2,6^ by *α* = −*γ*, where *γ* is the nonlinear selection gradient^20^ estimated from an individual fitness surface (that is, our estimate of selection given above). The stationary (deep-time) variance among lineages under such a process is given as $\frac{G}{{N}_{\rm{e}}}\times \frac{1}{2\alpha }$, which can be recovered under simulation by rescaling the Drosophilidae phylogeny from millions of years to units of generations (Extended Data Fig. 7). Stationary variances under the estimated value of stabilizing selection and *G* are plotted against a range of effective population (*N*_e_) sizes in Fig. 2. We find that our estimate of selection readily explains the constrained divergence in wing shape across the clade; the value of *N*_e_ necessary to produce an expected stationary variance consistent with the observed^11^ among-species phenotypic variance, given our estimate of selection, is low (*N*_e_ = 140, with an upper confidence limit approaching 5,000) but consistent with non-model wild *Drosophila* (for example, harmonic mean of estimates from *Drosophila buzzatii* = 174; ref. ^22^) as well as *D. melanogaster* evolve-and-resequence estimates (harmonic mean = 206; ref. ^23^), although inconsistent with some estimates of *D. melanogaster* *N*_e_ exceeding 1 million (ref. ^24^). For context, an *N*_e_ of nearly 20 million would be required in the absence of any stabilizing selection. This analysis can be seen as a proof of concept demonstrating that the observation of low divergence among species coupled with significant standing variance within species can be explained by observable levels of stabilizing selection.

Our results reconcile some aspects of fly wing evolution, yet also highlight some lingering challenges. First, our selection estimates cannot explain why some dimensions of wing shape exhibit conserved levels of high variance whereas others exhibit low variance. Although conserved stabilizing selection could explain such a pattern, it would lead to the opposite pattern across principal components of wing shape to that which we observed, with the weakest selection observed to act on the components with the most standing variance^25,26^. It is more likely^27^ that aspects of development play a leading role in the conserved eigen structure of wing variance^8,9^. Second, although a simple OU model parameterized with our estimate of total selection can reign in predicted divergence among species to the levels observed in nature, this model is clearly not a complete or accurate description of wing evolution. It seems untenable that all flies have evolved to a single global optimum wing shape, and the short phylogenetic half-life (4,016 generations, a miniscule fraction of the total depth of the Drosophilid phylogeny) is inconsistent with the observation^11^ of moderate to high phylogenetic signal in wing shape. Finally, *N*_e_ on the order of hundreds to ∼5,000 individuals is required to predict among-species variance congruent with observed values; although as noted above this is consistent with some recent estimates of *N*_e_ from structured wild populations and inferred from evolutionary response in the laboratory, it is inconsistent with other estimates^24^ indicating that global *N*_e_ can be very large for *D. melanogaster*. It is unclear what a reasonable long-term mean value of *N*_e_ would be for the clade. These last three problems could be resolved by a multi- or fluctuating-optimum^3^ OU process, which would lead to increases in predicted among-species variance at a given *N*_e_ and also increase phylogenetic signal. However, we lack any first principles for how optima should be placed or move on an adaptive landscape^28^ (but see ref. ^29^).

Stabilizing selection on wing shape in our experiment was driven almost entirely by males, which also exhibited greater variance in fitness than females (Levene’s test *F*_1,1804_ = 728, *P* < 0.0001). This finding of sex-specific selection explains the discrepancy between our findings of pervasive stabilizing selection and those of ref. ^9^, who found no evidence of stabilizing selection on the same wing landmarks in the distantly related but phenotypically similar *Sepsis punctum*; their analysis focused only on female fitness, rather than competitive male reproductive success. Although typically viewed as a diversifying force^30^, our results demonstrate that sex-specific selection can act as a force constraining the evolution of phenotypic diversity.

By inferring stabilizing selection in one species and relating our estimate to deep-time divergence across the entire clade, our study provides one empirical step towards reconciling the paradox of macroevolutionary stasis. Given that our estimate of stabilizing selection is approximately one-half the strength of the average value of nonlinear selection reported in studies of wild plants and animals^31^, this suggests that observable levels of stabilizing selection may often be able to reconcile low deep-time divergence in many taxa.

## Methods

Flies were *D. melanogaster* of the Larry Harshman moderate density (LHm) background. This lineage of flies was collected in California in the early 1990s^32^ and has been maintained since the mid-1990s at large, density-controlled population size under 25 °C temperature and standard *Drosophila* food media. This lineage is laboratory-adapted (over 500 generations in the laboratory) and harbours substantial genetic and phenotypic variance in most traits, including fitness.

### Fitness assay

We conducted a competitive fitness assay in which a focal, wild-type, unmated, male or female fly was placed in a vial with two other unmated flies, a male and female homozygous for the *bw* eye-colour mutation but otherwise of LHm background. Thus each focal fly was placed with a brown-eyed competitor of the same sex, as well as a brown-eyed mate of the opposite sex. Following 2 days of mating interaction, all flies were removed and resulting offspring were allowed to develop and scored for eye colour after eclosion. We thus obtained a binomial measure of reproductive success for each focal fly, although we also present an analysis of raw fecundity (see below). Our measurement of fitness via competitive reproductive success over a 2-day window is generally consistent with lifetime reproductive success in the context of propagation history of the LHm lineage, where the offspring founding a new generation are obtained after 18 h of egg laying following 2 days of mating interaction. The fitness assay was blocked by generation (*n* = 8) and conducted at room temperature for logistical reasons.

### Wing phenotyping

Following the mating assay, focal flies were frozen, and later the right wing (left, if the right was damaged) was removed, dry mounted under a standard cover slip and photographed under transmitted light using a stereomicroscope (Olympus SZX-10). The microscope was fitted with an objective revolver allowing the objective to be aligned with the photographic optical path, eliminating the distortion otherwise inherent in imaging through a stereomicroscope. A stage micrometre was photographed following the same procedures to scale landmark measurements to units of mm. The 12 vein-intersection fixed landmarks were then placed by hand using geomorph^33^ in R. Severely damaged wings or those missing cross veins were excluded, although we did include a few wings which had a missing landmark; for these we used a thin-plate spline procedure to estimate the missing landmark using geomorph. Landmark coordinates were rescaled from units of raw pixels to millimetres, using the mean of measures from separate images of a stage micrometre imaged identically to the wings. We then used a Procrustes analysis using Morpho^34^ in R to scale and rotate the landmark coordinates to obtain standardized shape variables, using after ref. ^11^ the *D. melanogaster* consensus as a target in the Procrustes rotation. We multiplied the resulting raw shape variables by 1,000 to be consistent with past work^11^. To estimate the phenotypic covariance matrix to perform a principal component analysis, we used a Bayesian multiresponse mixed effects model with the vector of shape traits as the response, sex of the fly as a fixed effect and block as a *g*-side random effect; the *r*-side unstructured covariance matrix gives an estimate of *P*. We assumed non-informative priors for fixed and random effects. We projected eigenvectors of *P*, as well as previous estimates (from ref. ^11^) of *G*, heterozygous *M* (conclusions the same for homozygous estimate) and *R*, on the centred shape data to obtain phenotypic scores on the principal components of these matrices. Note that as a result of scaling and rotation inherent in the Procrustes analysis, there are 20 non-null dimensions of shape variation each of these covariance matrices. Our estimate of *P* was quite similar to the other covariance matrices; matrix comparison based on Krzanowski’s common subspace approach^35,36^ indicated that *P* shared nearly 90% of the subspace defined by the first 12 principal components when compared with *G* (89%), with *R* (84%) and *M* (76%).

### Statistical analysis

After excluding flies for which no offspring were produced by either focal or competitor fly in the fitness assay, we obtained complete fitness and phenotypic records for 1,806 flies. We estimated unstandardized (that is, no trait standardization, absolute fitness) quadratic selection differentials, conditioned on sex, separately for each principal component by fitting separate generalized mixed effects modes each with sex as a fixed effect, as well as linear and quadratic terms for PC score. Block was included as a random effect. The response variable was the vector of red-eyed and brown-eyed offspring number. We assumed a binomial distribution for the response variable, and estimated the residual variance (instead of fixing to unity), as the response was not a single Bernoulli trial but rather many for each data point, and so overdispersion is possible (even likely)^17^. We then approximated^17,37^ the nonlinear selection differential^26^ *C* from the quadratic regression coefficient (*c*) as $C=P[(\bar{\hat{p}}(1-\bar{\hat{p}})2c]P$ (where $\bar{\hat{p}}$ is the mean predicted value of individual fitness), performing this across the posterior distribution to obtain credible intervals. We also report estimates of variance-standardized quadratic selection differentials in Extended Data Fig. 2. We then fitted linear regressions of the sign-log-transformed quadratic selection differentials on log-transformed phenotypic standard deviation of each principal component (taken as the square root of the variance of phenotypic scores on the component vector), where the sign-log transform of *C* is ${\rm{sign}}\left(C\right)\times\mathrm{ln}({|C|}+1)$. This transformation, which has the desirable property that zeros are retained as zero, allows the fitting of a log-log regression to assess proportional relationships between selection and variation, given that our measures of selection are often negative and span a range of both positive and negative values.

To understand the null expected relationships between phenotypic variance and the strength of selection, we performed a simulation in which we used our observed fitness values and simulated trait values with varying degrees of phenotypic variance, with 1,000 levels ranging from the observed minimum and maximum standard deviations across principal components of *P*. For each level we performed 1,000 iterations, where for each iteration we sampled a trait *x* from a normal distribution centred at zero, and then calculated cov(*w*, *x*^2^), where we first centred *w* by sex and block. This generated a null distribution of quadratic selection differentials, where the expected covariance is zero (no selection), across the range of phenotypic variance we observed. We then plotted the 95% probability band obtained from quantile regression of the simulated *C* values on the phenotypic standard deviation.

We estimated selection on total Euclidean distance by fitting a binomial GLMM with red- and brown-eyed offspring number as the response, sex as a fixed effect, as well as the sum of individual PC scores and the sum of squared PC scores as fixed effects. The sum of raw PC scores is necessary to account for any directional selection (although we found no statistically significant directional selection in this analysis), the sum of squared PC scores is a measure of the squared Euclidean distance from the mean wing shape. We re-fit this model to include an interaction with sex and the other fixed effects, to test the hypothesis that selection may be sex specific. We obtained the same conclusions when fitting a similar model, but with the number of red-eyed offspring a Poisson response variable. To obtain a Lande–Arnold^20^ estimate of nonlinear selection on the total phenotypic variance for use in an OU model that is most appropriate for approximating the adaptive landscape^38^, we fitted a linear mixed model with the sum of raw PC scores and squared Euclidean distance as fixed effects. We modelled absolute fitness as a Gaussian response variable, including sex as a fixed effect to account for sex differences in mean fitness^39^. Total offspring number was included as a weight term. We then used two times^40^ the estimate of the nonlinear regression term, divided by mean absolute fitness, as our estimate of *γ*; this gave us a classical Lande–Arnold^20^ estimate of nonlinear selection, pooled by sex, that avoids the challenges^17^ of approximation of nonlinear selection gradients from a glm regression coefficient.

To assess fitness effects of missing cross veins, we fitted a binomial mixed effects model to an expanded dataset that included flies with missing cross veins. These flies were excluded from the landmark analysis as all of the landmarks could not be placed on these mutant individuals. Our model included wing type (wild-type or cross-vein mutant) as a fixed effect. We plot the data (Extended Data Fig. 4) using the log odds of fitness (ln(red + 1/brown + 1)) as this is easier to visualize.

For our assessment of OU model fits, we obtained an estimate of total within-species genetic variance and among-species phenotypic variance as the sums of the diagonals of the *G* and *R* (respectively) matrices for wing shape obtained from ref. ^11^, as well as our own estimate of *α* (see above). We confirmed the stationary expectation for the OU process by simulating 1,000 runs of OU evolution assuming an *N*_e_ of 140, calculated as the *N*_e_ required to produce a theoretical stationary variance equivalent to the observed among-species variance (5,487) given our estimate of selection. Simulations (Extended Data Fig. 5) were performed in phytools^41^, using the a time-calibrated Drosophilid phylogeny obtained (by vcv2phylo() in ape^42^) from the phylogenetic covariance matrix presented in ref. ^11^. We rescaled the branch lengths from millions of years to generations assuming ten generations per year for wild *Drosophila*. We based this generation time on inferred values of 15 and 3–5 generations per year for wild *D. melanogaster*^43^ and *Drosophila obscura/subobscura*^44^, respectively, but note that conclusions do not depend on the details of this assumption because of the depth of the tree.

#### Assessment of size effects

We focus on analysis of wing shape traits as the conserved variance in these shape components presents a primary unsolved puzzle. However, we explored the effects of including the natural logarithm of wing centroid size (both as linear and quadratic terms) in all of our selection models, and in all cases conclusions remain unchanged. Across principal components of phenotypic variation, we find persistent stabilizing selection on wing shape traits when including log centroid size and its quadratic term in the mixed models used to estimate *C* (Extended Data Fig. 6). Moreover, when assessing selection on total Euclidean distance from the mean, we find there remains statistically significant selection on squared Euclidean distance when including log centroid size and its quadratic term in the model (GLMM coefficient for squared Euclidean distance = −0.00099, CI = [−0.00163, −0.00049], *P*_mcmc_ = 0.006).

All analyses were performed in R and reported statistical tests are two-sided. Mixed models were fitted by Bayesian Markov chain Monte Carlo (MCMC) in MCMCglmm^45^, with the exception of the linear mixed model used to estimate *γ* which was fitted by REML using lme4^46^. Large language models (ChatGPT) were used as a coding aid in modifying R code for producing visually appealing figures for some of the figures.

### Reporting summary

Further information on research design is available in the Nature Portfolio Reporting Summary linked to this article.

## Supplementary information

Reporting Summary

Peer Review File

## Data Availability

Data to reproduce all results and figures are available via Zenodo at 10.5281/zenodo.20338922 (ref. ^47^).

## Code availability

Codes to reproduce all results and figures are available via Zenodo at 10.5281/zenodo.20338922 (ref. ^47^).
