# Supplementary material for: Apparent stabilizing selection explains micro- and macroevolution of Drosophila wings
Source: Nat Ecol Evol. 2026 Jul 10;10(8):1413–8. doi: 10.1038/s41559-026-03121-2 (PMC13441934; doi:10.1038/s41559-026-03121-2)
Supplement: Supplementary file 2 [file 41559_2026_3121_MOESM2_ESM.pdf]

nature portfolio

Peer Review File

Apparent stabilizing selection explains micro and macroevolution of Drosophila wings

Corresponding Author: Dr Stephen De Lisle

Version 0:

Decision Letter:

5th March 2026

Dear Stephen,

Soon after I sent you our decision letter, Reviewer #2 informed us about a typo in their review report. I have copied their message below. Please take this into consideration when responding to their comments:

In last sentence of the first paragraph of comment #3, it was written "[low evolutionary divergence relative to neutral expectation] is an artifact like the scaling relationship", but it should have been "[low evolutionary divergence relative to neutral expectation] is not an artifact like the scaling relationship". The "not" is missing in the submitted report.

Please accept my apologies for the inconvenience, and feel free to let me know if you have any concerns at any time. I look forward to seeing the revised manuscript.

[redacted]

Reviewer expertise:

Reviewer #1: phenotypic evolution, macroevolution, quantitative genetics

Reviewer #2: evolutionary theory, phenomics

Reviewers' comments:

Reviewer #1 (Remarks to the Author):

This is an important contribution toward resolving the puzzle of apparent stasis in wing shape evolution - a puzzle that extends well beyond fly wings. Although the study does not fully resolve the issue, as the authors appropriately acknowledge, it is nevertheless interesting, thought-provoking, and potentially far-reaching in its implications.
However, I believe there are several major limitations. Specifically, I am not convinced that (i) the estimated Ne values are biologically "reasonable" (line 38), (ii) it remains unclear how size, allometry, and selection on size are incorporated into the analyses and interpretation, and (iii) I do not fully follow the argument surrounding the cross-veinless phenotype. If these points can be clarified and strengthened, I believe the manuscript would make a strong contribution.

1. Ne
As I understand it, simulations assuming an Ne of 142 generate a theoretical stationary variance comparable to the observed among-species variance. Although the confidence intervals are large, the point estimate itself appears very low. Are these effective population size estimates truly biologically "reasonable", as stated on line 38? To me, the reported Ne values seem extremely low for drosophilids. D. melanogaster is typically inferred to have substantially higher effective

population sizes. Is there strong empirical evidence from other Drosophila (or other dipteran species) supporting effective population sizes in this range? If so, this should be made explicit. I consider the plausibility of Ne critical for justification and the overall conclusions

2. Cross-veinless "major-effect mutation"
The arguments around the crossveinless phenotypes are interesting, but I am not convinced that this is a genetic phenomenon. The absence of crossveins can result from mutations, but it is likely to represent a non-genetic aberration. Lack of crossveins (including asymmetry e.g., missing only on the left or right wing) is relatively common in lab strains and crossveins are well-known to fail to form under environmental stress (as in the classic Waddington experiments). Is this truly a genetic effect? If so, could it reflect somatic mutations? And if it is somatic, how does that inform the pleiotropic landscape? Given this, I am not convinced that the observed crossveinless phenotypes necessarily represent major-effect genetic mutations. This severely weakens the argument that the lack of detectable selection on these individuals provides strong evidence for pleiotropic stabilizing selection. However, I still think that the overall argument in that paragraph holds without this third line of evidence.

3. Statistical treatment of size and allometry
It is unclear to me how selection on overall body size and wing size (which itself is almost guaranteed to be stabilizing) impacts selection on wing shape variables. Procrustes analysis brings all variables to the same scale, but it does not remove the covariance between (centroid) size and shape. Allometric shape variation is thus still part of the wing shape data and it is unclear to me how the analysis takes selection on body (and relative wing) size into account? This is unclear but is critical. If wing shape allometry is not taken into account, I actually do not follow the main argument.
As a related point: Early in the manuscript, it would help to be explicit about which traits are included as covariates in the selection analysis (i.e., being explicit whether this is direct or indirect selection after taking size into account).

Other comments:
- Could the decrease in the strength of stabilizing selection with increasing rank of the eigenvectors be explained by measurement error or limited statistical power to detect strong stabilizing selection when studying traits with less variance (and more relative error)?
- How similar is the original eigenvector structure compared to Houle et al.'s? How comparable are the leading axes? I realize there may not be a better approach, but a more explicit comparison would help convince more critical readers that the axes are meaningfully aligned and that the major PCs in the Houle matrix are at least similar to the PCs in the new data.
- I agree that the experimental setup likely does not impose strong selection on wings. However, to what extent can these results be interpreted ecologically? The population is lab-adapted and fitness is measured under laboratory conditions. How do these patterns relate to natural field conditions?
- Line 275: please provide a reference for the estimated number of generations of drosophilids per year. Readers will disagree almost any number, so I think this needs a citation. I also expect that the true number of generations must vary a lot both within and among species.
- If possible, I suggest adding a stronger, more integrative concluding paragraph that would help emphasize the broader implications and leave the reader with a clearer take-home message.
- For Figure 2, consider plotting the selection coefficient against the proportion of variance explained for each matrix rather than against eigenvector rank. The rank order will inevitably be similar across matrices, but the matrices differ in their levels of integration (eccentricity), and this alternative visualization might better capture those differences.
- In panel B (which is stunning!), please move the legend so that it does not overlap with the data.

Reviewer #2 (Remarks to the Author):

In this study, the authors measured stabilizing selection acting on the wing morphology of fruit flies, utilizing a new dataset with fitness measurements. They found various wing shape traits to be under stabilizing selection and associated with male reproductive fitness. In addition, strength of stabilizing selection was positively correlated with phenotypic variance. The authors suggested that the stabilizing selection is likely apparent, resulting from pleiotropy.

This study complements previous work with new data and analyses, and offers valuable insights into observed evolutionary patterns that have been deemed difficult to explain. Together, I will be happy to see these new results published. I do have some comments on this manuscript, but they are mainly about the presentation of the results and how they might be better connected to contexts of earlier studies.

1. Relationship between strength of selection and standing variances
In the text, the result is first described qualitatively as "[estimates of quadratic selection differentials are] related strongly to the amount of phenotypic variance" (line 85), which I can see in Fig. 2A. It is also stated that "the amount of stabilizing selection is proportional to the amount of variance in any given combination of wing shape traits" (line 90-92). It is not clear from Fig. 2A, however, if the relationship is "proportional", as the X-axis is rank order of principal components rather than variance. To this end, I suggest including plots with variances as X-axes. It is also desirable to have a statistic that describes the relationships (e.g., correlation coefficient) to give more support for the verbal description. (Although the "proportionality" would be "verified" by another analysis, that part has clarity issues of its own; see below.)

It is also stated that "the same pattern" was found when selection was estimated from phenotypic scores on G, M, and R

matrices (line 86-89). In Fig. 2A, however, there appears to be some difference despite qualitative similarity. The patterns, presumably, should not be considered “the same” or, if the authors do consider the apparent similarity is trivial, some explanation is needed.

2. Model of selection on total phenotypic deviation from mean
The “proportionality”, which is not obvious in Fig. 2A, is interpreted as consistent with a model of selection on overall distance from the mean (optimum) (line 91-93). The interpretation was tested by fitting a model of selection on total squared Euclidean distance and is supported (Fig. 2B). It is not shown, however, if the said model is indeed expected to produce the said pattern. The authors essentially state the regime of selection that is confirmed by model fitting (and presented in Fig. 2B) is expected to produce (at least some) observations shown in Fig. 2A, yet it is largely unexplained why there is such an expectation. In Methods, I only found how strength of selection was estimated (line 227-238) and how the model fitting was done (line 240-258), but not theoretical expectations/predictions that would bridge the two results.

If the expectation comes from previous theoretical studies, they need to be cited (in line 93). If the authors derived it, I would suggest presenting some formulation in Methods and/or some simulation results as an extended figure. If this model of selection is effectively embedded in pre-existing evolutionary models but the prediction about the variance-selection relationship has not been explicitly presented, I would also recommend adding some details to Methods.

In addition, it is not said explicitly either if the “amount of variance” in line 91 refers specifically to phenotypic, mutational, or genetic variance; it is thus unclear what exactly the model’s prediction is. Is strength of selection expected to be proportional to phenotypic, mutational, or genetic variance? Are these variances also proportional to each other under this model? Clarification would be desirable.

3. Connection to previous analyses of fly wing traits
In an introductory paragraph, the authors mentioned results of Houle et al. (2017). However, the reported similarity between the M matrix and G or R matrices, which is mentioned in line 71-74, is exaggerated due to their biased method for matrix comparison; nevertheless, there is still significant alignment between them (Jiang and Zhang, 2020). I suggest this issue being mentioned as long as this very result of Houle et al. (2017)’s is being mentioned. Houle et al. later proposed an alternative way of matrix comparison in a preprint, but in the same manuscript they also acknowledged that their original method is not informative (Houle et al., 2020). The observation of very low evolutionary divergence relative to neutral expectation, which appears to be a motivator of this study, is an artifact like the scaling relationship, so it may be desirable to also clarify that the present study is “safe”.

The scaling relationship between mutational variances and evolutionary rates of wing shape traits reported in Jiang and Zhang (2020), which was interpreted as that phenotypic dimensions with more mutational variance were under stronger (apparent) stabilizing selection, also appear consistent with the finding of this study. For this connection, I suggest that the previous result is mentioned, maybe in the sentence of line 112-117.

The reanalysis mentioned above (Jiang and Zhang, 2020) proposed an explanation for fly wing evolution that is essentially apparent stabilizing selection. They demonstrated using simulations that such a model can reproduce observed patterns, but could not directly test if the same mechanism did contribute to fly wing evolution (which is made possible by new data). Thus, I suggest that it is briefly mentioned, perhaps near the sentence of line 105-106.

Minor comments
Line 77-79: While the cited paper did use the word “evolvability” to refer to additive genetic variance (though with phenotypic variance used as a proxy), this is not a practice I would recommend, personally. I consider evolvability as a property that is affected by many factors including genetic and phenotypic variances and thus not a synonym of the latter. Thus, I would recommend using “additive genetic variance” or “phenotypic variance” here instead.

Line 152-154: Cheverud (1984) would be relevant to cite here, as it was an early theory paper that connected variation to developmental constraints.

Line 166-167: I agree that, in general, we cannot predict the movement of adaptive peaks very well due to the lack of first principles. There has been some hypothesis about this though, and the idea of a “selection line of least resistance” may be relevant to cite here (Arnold, 2023; the idea can be seen in his earlier papers, but I’m only citing his recent book here for convenience).
I find it a bit difficult to read Fig. 2A with four sets of data points put together and three of them (G, M, R) in somewhat transparent color. I would prefer them being plotted separately and placed side by side.

I think it is better to note that “glmm” means “generalized linear mixed model” when it appears for the first time (line 96), in case readers get confused. I also suggest writing abbreviations like GLMM in capital letters for better readability.

Note that I did not take format requirements that might exist into account when making suggestions. Apologies for any inconvenience this might cause.

References
Cheverud J. M. (1984) Quantitative genetics and developmental constraints on evolution by selection. J. Theor. Biol.
Jiang D. and Zhang J. (2020) Fly wing evolution explained by a neutral model with mutational pleiotropy. Evolution.

Houle D., Bolstad G. H., Hansen, T. F. (2020) Fly wing evolutionary rate is a near-isometric function of mutational variation. BioRxiv.
Arnold S. (2023) Evolutionary Quantitative Genetics. (book)

********************END********************

Version 1:

Decision Letter:

24th April 2026

Dear Stephen,

Thank you for submitting your revised manuscript "Apparent stabilizing selection explains micro and macroevolution of Drosophila wings" (NATECOLEVOL-26010090A). It has now been seen again by the original reviewers and their comments are below. The reviewers find that the paper has improved in revision, and therefore we'll be happy in principle to publish it in Nature Ecology & Evolution, pending minor revisions to satisfy the reviewers' final requests and to comply with our editorial and formatting guidelines.

If the current version of your manuscript is in a PDF format, please email us a copy of the file in an editable format (Microsoft Word or LaTex)-- we can not proceed with PDFs at this stage.

If you have not done so already, please ensure that you also email us a completed copy of the Reporting summary :

Reporting summary: https://www.nature.com/documents/nr-reporting-summary.pdf

Next steps: We will now perform detailed checks on the current version of your paper and send you a checklist of our editorial and formatting requirements in about a week. Meanwhile, you can start revising the manuscript to address the remaining comments from reviewers, but please do not upload any final materials to the submission system until you receive the formatting checklist from us. Once you receive the checklist, you can incorporate the formatting changes in the revised manuscript. Then, you can upload the final materials to the submission system.

Thank you again for your interest in Nature Ecology & Evolution. Please do not hesitate to contact me if you have any questions.

[redacted]

Reviewer #1 (Remarks to the Author):

I would like to thank the Authors for their careful revisions to the manuscript. My previous concerns regarding size and allometry have been fully addressed through the additional analyses and responses provided.

I do, however, find the discussion of effective population size (Ne) somewhat unclear (or incomplete). In particular, I think it's important to note in the main text that some published estimates exceed one million. The current version simply states the estimates in melanogaster are "very large". I suspect that most readers will find the numbers presented in the manuscript to be small, and being upfront about the range of estimates will make this more transparent. I don't disagree with the Authors' conclusions, but providing a more explicit and balanced range of estimates seems appropriate.

I have no further comments or suggestions beyond this point, aside from recommending a check of the spelling of “Ornstein–Uhlenbeck” (line 143).

Reviewer #2 (Remarks to the Author):

I greatly appreciate what the authors have done to address my questions. I think this version of the manuscript is overall strong and well written. In particular, the updated Figure 2 is a lot more informative now. I only have some minor comments for this version.

1. While Fig. 2A is shrunk, I agree that the newly added Fig. 2B is helpful and more than made up for it. It is now shown that the four regression slopes are not significantly different from -1. I think it would also be worth it to mention in the main text that the four CIs also overlap, which would strengthen the statement that the four slopes are “similar” to each other. Hopefully

this only adds one more sentence.

Note that the figure in the PDF file I can access now has much lower resolution than the original version's PDF; the figure in the marked Word document is fine.

2. In the main text, where X-axes of Fig 2A-B are referred to simply as "phenotypic score on [P, M, G, or R matrix]" (line 89). It is not entirely clear, given the main text alone, how these scores were obtained and how they differed from the "original" scores. Thus, it might be better to say "phenotypic score on principal components [of P, M, G, or R matrix]" instead (as in Methods, line 276), which I think is clearer and would not add too much more text. Alternatively, the result can be described as "relationship between selection differential and mutational/genetic/evolutionary variance", as in line 86.

I should have brought this up in the last round of review since this is not a new issue that arose in the revision. Apologies for any inconvenience.

3. Line 275: It is added that it is the "heterozygous" M-matrix (the matrix denoted as M_het in Houle et al., 2017, supposedly?) that was used. I appreciate the clarification, but also wonder why they would like to focus on M_het instead of M_hom (e.g., Houle et al. put more emphasis on M_hom and presented it as a "main" result).

Open Access This Peer Review File is licensed under a Creative Commons Attribution 4.0 International License, which permits use, sharing, adaptation, distribution and reproduction in any medium or format, as long as you give appropriate credit to the original author(s) and the source, provide a link to the Creative Commons license, and indicate if changes were made.
In cases where reviewers are anonymous, credit should be given to 'Anonymous Referee' and the source.
The images or other third party material in this Peer Review File are included in the article's Creative Commons license, unless indicated otherwise in a credit line to the material. If material is not included in the article's Creative Commons license and your intended use is not permitted by statutory regulation or exceeds the permitted use, you will need to obtain permission directly from the copyright holder.
To view a copy of this license, visit https://creativecommons.org/licenses/by/4.0/

Reviewer #1 (Remarks to the Author):

This is an important contribution toward resolving the puzzle of apparent stasis in wing shape evolution - a puzzle that extends well beyond fly wings. Although the study does not fully resolve the issue, as the authors appropriately acknowledge, it is nevertheless interesting, thought-provoking, and potentially far-reaching in its implications.
However, I believe there are several major limitations. Specifically, I am not convinced that (i) the estimated Ne values are biologically “reasonable” (line 38), (ii) it remains unclear how size, allometry, and selection on size are incorporated into the analyses and interpretation, and (iii) I do not fully follow the argument surrounding the cross-veinless phenotype. If these points can be clarified and strengthened, I believe the manuscript would make a strong contribution.

We thank the reviewer for their constructive concerns, and have revised our manuscrip to address each, which we adress in detail below.

1. Ne
As I understand it, simulations assuming an Ne of 142 generate a theoretical stationary variance comparable to the observed among-species variance. Although the confidence intervals are large, the point estimate itself appears very low. Are these effective population size estimates truly biologically “reasonable”, as stated on line 38? To me, the reported Ne values seem extremely low for drosophilids. D. melanogaster is typically inferred to have substantially higher effective population sizes. Is there strong empirical evidence from other Drosophila (or other dipteran species) supporting effective population sizes in this range? If so, this should be made explicit. I consider the plausibility of Ne critical for justification and the overall conclusions

First, to clarify one point regarding the reviewers first sentence here, we performed OU simulations simply to confirm that stationary variance is achieved over the timescale in question. That step is really more of a confirmation, and is perhaps not strictly neccisary given that the phylogeny is quite deep. We present it simply for thoroughness.

But the reviewer is correct- the value of Ne needed to explain among-species divergence, *given our estimate of stabalzing selection*, is quite low (updated to 140 now in the revised version, with an upper 95 CI of approx. 5000. We re-fit the selection model using a slightly different approach [absolute fitness as response, sex as a fixed effect, and then standardizing the resulting gradient by mean fitness]; this is a more statistically justified approach than separate fitness relativizations for each sex prior to fitting the regression. Regardless the main conclusions are the same)

Whether this is completely unreasonable, or well within expections for actual populations for flies evolving in the wild, is unclear. For example, in wild D. buzzati, Barker 2011 reports a range of estimates with a harmonic mean of Ne = 174. In evolve and requequence experiments with D. melanogaster, Jónás et al. 2016 Genetics report a harmoic mean of Ne = 206. Yet, Sprengelmeyer et al. 2020 MBE report continent wide genomic estimates of over 1 million for african D. melanogaster. So there seems to be substantial variation among estimates of *Drosophila* Ne. Our inference is in line with the lower estimates, but noteworthy is that these lower estimates typically come from more realistic biological context than, for exmaple, contintent wide genomic data.

We now report all of the above in the main text (Line 160-172, 191-206). We are not going out on a limb to try to make a bold statement about Ne in *Drosophila*, but rather that our estimate is within the range of *some* reported estimates. Our interpretation of this is that an Ne of 140 (with an upper bound near 5000) is perhaps not completely unreasonable, in the context of a clade-wide harmonic mean Ne. Yet we aknowledge it is not entirely clear what a reasonable value is, and note several factors that could reconcile our estimate of selection, observed among species divergence, and Ne.

For context, consider that in the absence of selection, an Ne of approximately 20 million would be needed to reconcile observed levels of genetic variation with observed among species divergence (now noted in the manuscript, Line 171-172).

2. Cross-veinless “major-effect mutation”
The arguments around the crossveinless phenotypes are interesting, but I am not convinced that this is a genetic phenomenon. The absence of crossveins can result from mutations, but it is likely to represent a non-genetic aberration. Lack of crossveins (including asymmetry e.g., missing only on the left or right wing) is relatively common in lab strains and crossveins are well-known to fail to form under environmental stress (as in the classic Waddington experiments). Is this truly a genetic effect? If so, could it reflect somatic mutations? And if it is somatic, how does that inform the pleiotropic landscape? Given this, I am not convinced that the observed crossveinless phenotypes necessarily represent major-effect genetic mutations. This severely weakens the argument that the lack of detectable selection on these individuals provides strong evidence for pleiotropic

stabilizing selection. However, I still think that the overall argument in that paragraph holds without this third line of evidence.

The reivewer has a good point that the crossveinless phenotype could be an environmental effect (and we now point this out in the manuscript). However, rather than undermine our argument that the seleciton we observe is likely apparent, this in fact strengthens it.

IF selection acted directly on wing shape / vein structure, we would expect crossveinless to have a fitness effect even if it was environmentally induced. In fact, environmental manipulation of phenotypes is a classical approach for demonstrating a trait is a direct target of selection (e.g., think of the tail feather manipulations in Malte Andersson's widow birds). The fact we observe no fitness effect of these phenotypes, given that they are likely either an environmental effect or result of a major effect mutation directly affecting wing structure, suggests that the relationship between quantitative variation in wing landmarks and fitness is probably an outcome of plieotropic effects on other traits that directly effect fitness. However, we readily admit that this is not decisive proof, and believe we have worded our argument conservatively.

We have modified the text to clarify all of the above points. (line 138-141)

3. Statistical treatment of size and allometry
It is unclear to me how selection on overall body size and wing size (which itself is almost guaranteed to be stabilizing) impacts selection on wing shape variables. Procrustes analysis brings all variables to the same scale, but it does not remove the covariance between (centroid) size and shape. Allometric shape variation is thus still part of the wing shape data and it is unclear to me how the analysis takes selection on body (and relative wing) size into account? This is unclear but is critical. If wing shape allometry is not taken into account, I actually do not follow the main argument.
As a related point: Early in the manuscript, it would help to be explicit about which traits are included as covariates in the selection analysis (i.e., being explicit whether this is direct or indirect selection after taking size into account).

The resuts we present are from an analysis of wing shape alone (although controlling for block and sex). We had originally explored effects of centroid size and its quadratic term, and inclusion of these terms did not change conlcusions regarding selection on wing shape, and so we did not present them. Below we justify this decision. However, we appreciate the reviewers concern and now present results from models that include log centroid size (linear and quadratic terms). We present both estimates of quadratic differentials across each principal component of wing shape (Extended data figure 4), as well as report selection (which remains stabalizing and staitstically signifcant) on total squared Euclidean distance in wing shape while including log centroid size and its quadratic term. We did not find pervasive stabalziing selection on centroid size, although there is directional selection on size.

Conceptually, we beilieve that the most interesting aspect of fly wing evolution is conserved patterns of wing shape variation, rather than size itself. Because we are assessing and interpreting what we suspect is apparent, rather than direct, selection, we readily embrace the fact that we have not included all of the traits causally affecting fitness. If we were interpreting our results as evidence that wing shape causily affects fitness, we agree with the reviewer it would be extrememly important to include size (although as we point out, it actually does not change conclusions)

Besides presenting the additional analyses including size, we have also modified our main text in a couple of places to clarify these points: 117, 139-140, and also have added an extended discussion in the methods (line 389-400)

Other comments:
- Could the decrease in the strength of stabilizing selection with increasing rank of the eigenvectors be explained by measurement error or limited statistical power to detect strong stabilizing selection when studying traits with less variance (and more relative error)?

This is a good point, and to address this concern, we now present a simulation of the expected relationship between quadratic selection and phenotypic variance under the null of zero selection (Extended data figure 2). In summary we do expect variation in the estimates to relate to trait variance, but as we see in the simulation the pattern we observe in our data departs from the null in meaningful ways- first that the estimates are nearly always negative (selection is stabalizing), and the relationship with phenotypic variation (described more below in response to reviewer 2) diverges beyond the 95% limits of the null.

- How similar is the original eigenvector structure compared to Houle et al.'s? How comparable are the leading axes? I realize there may not be a better approach, but a more explicit comparison would help convince more

critical readers that the axes are meaningfully aligned and that the major PCs in the Houle matrix are at least similar to the PCs in the new data.

We now report matrix comparison statistics (Krzynawoski subspace comparison, which compares the subspace of two matrices defined by their higher ranking PCs) in the methods (line 278-281), showing that our P and Houle's G share nearly 90% of the same subspace defined by the first 12 PCs. M and R are 76% and 83%. So in summary our estimate of P is quite close to G, and also close (but not as close) to M and R.

- I agree that the experimental setup likely does not impose strong selection on wings. However, to what extent can these results be interpreted ecologically? The population is lab-adapted and fitness is measured under laboratory conditions. How do these patterns relate to natural field conditions?

As we state in our manuscript, we believe that apparent selection measured in a benign lab environment could represent (L 175) "a background force expected to constrain divergence." In our revised manuscript, we now relate our estimate to other estimates of selection in the wild, noting that our inferred measure of stabalizing selection is approximately half the strength of mean nonlinear selection reported in studies of wild populations (line 251-256)

- Line 275: please provide a reference for the estimated number of generations of drosophilids per year. Readers will disagree almost any number, so I think this needs a citation. I also expect that the true number of generations must vary a lot both within and among species.
We agree with these points. We based our value of 10 on published estimates of 15 and 3-5 for melanogaster and subobscura. Importantly, it doesn't really matter in terms of conclusions, because the phylogeny is deep enough that we expect that stationary variance of the OU process to be reached regardless. We now clarify this in the methods. (line 359-361)

- If possible, I suggest adding a stronger, more integrative concluding paragraph that would help emphasize the broader implications and leave the reader with a clearer take-home message.
We have added this:

"By inferring stabilizing selection in one species and relating our estimate to deep-time divergence across the entire clade, our study provides one empirical step towards reconciling the paradox of macroevolutionary stasis. Given that our estimate of stabilizing selection is approximately one-half the strength of the average value of nonlinear selection reported in studies of wild plants and animals28, this suggests that observable levels of stabilizing selection may often be able to reconcile low deep-time divergence in many taxa. "
- For Figure 2, consider plotting the selection coefficient against the proportion of variance explained for each matrix rather than against eigenvector rank. The rank order will inevitably be similar across matrices, but the matrices differ in their levels of integration (eccentricity), and this alternative visualization might better capture those differences.
This is closely related to reviewer 2's comment, and so we address it in depth below. In short we have now done this, plotting selection estimates against the phenotypic sd for each dimension.
- In panel B (which is stunning!), please move the legend so that it does not overlap with the data.
Corrected

Reviewer #2 (Remarks to the Author):

In this study, the authors measured stabilizing selection acting on the wing morphology of fruit flies, utilizing a new dataset with fitness measurements. They found various wing shape traits to be under stabilizing selection and associated with male reproductive fitness. In addition, strength of stabilizing selection was positively correlated with phenotypic variance. The authors suggested that the stabilizing selection is likely apparent, resulting from pleiotropy.

This study complements previous work with new data and analyses, and offers valuable insights into observed evolutionary patterns that have been deemed difficult to explain. Together, I will be happy to see these new results published. I do have some comments on this manuscript, but they are mainly about the presentation of the results and how they might be better connected to contexts of earlier studies.

We thank the reviewer for their comments and suggestions, and have done our best to address their concerns. In particular we would like to thank the reviewer for pointing us to Jiang and Zhang 2020, a key reference we had missed.

1. Relationship between strength of selection and standing variances
In the text, the result is first described qualitatively as "[estimates of quadratic selection differentials are] related strongly to the amount of phenotypic variance" (line 85), which I can see in Fig. 2A. It is also stated that "the amount of stabilizing selection is proportional to the amount of variance in any given combination of wing shape traits" (line 90-92). It is not clear from Fig. 2A, however, if the relationship is "proportional", as the X-axis is rank order of principal components rather than variance. To this end, I suggest including plots with variances as X-

axes. It is also desirable to have a statistic that describes the relationships (e.g., correlation coefficient) to give more support for the verbal description. (Although the "proportionality" would be "verified" by another analysis, that part has clarity issues of its own; see below.)

This is a good point, and similar to a suggestion made by reviewer 1. To address this, we now present (Figure 2B) regressions between the sign-log transformed selection differentials and log transformed phenotypic standard deviations for each principal component. The sign-log transform allows us to place the selection differentials (which are mostly negative) into log-log space. These regressions all yield slopes that differ signifcantly from zero but do not differ significantly from -1, or eachother. This provides statistical support for the conjecture we made about selection being proportional to variation in each dimension and similar across phentoypic scores on PCs from the different matrices (which we discuss at line 91-93)

It is also stated that "the same pattern" was found when selection was estimated from phenotypic scores on G, M, and R matrices (line 86-89). In Fig. 2A, however, there appears to be some difference despite qualitative similarity. The patterns, presumably, should not be considered "the same" or, if the authors do consider the apparent similarity is trivial, some explanation is needed.

We have changed the wording here ("similar") and now also present the regressions mentioned above, which indicate that the relationships between phenotypic variation and selection is indeed similar regardless of which matrix is used to rotate the data.

2. Model of selection on total phenotypic deviation from mean
The "proportionality", which is not obvious in Fig. 2A, is interpreted as consistent with a model of selection on overall distance from the mean (optimum) (line 91-93). The interpretation was tested by fitting a model of selection on total squared Euclidean distance and is supported (Fig. 2B). It is not shown, however, if the said model is indeed expected to produce the said pattern. The authors essentially state the regime of selection that is confirmed by model fitting (and presented in Fig. 2B) is expected to produce (at least some) observations shown in Fig. 2A, yet it is largely unexplained why there is such an expectation. In Methods, I only found how strength of selection was estimated (line 227-238) and how the model fitting was done (line 240-258), but not theoretical expectations/predictions that would bridge the two results.

Regarding the first point about proportionality, see our reply to the previous comment. We have now fit log-log regressions which let us assess this statement directly.

If the expectation comes from previous theoretical studies, they need to be cited (in line 93). If the authors derived it, I would suggest presenting some formulation in Methods and/or some simulation results as an extended figure. If this model of selection is effectively embedded in pre-existing evolutionary models but the prediction about the variance-selection relationship has not been explicitly presented, I would also recommend adding some details to Methods.
See response to the next comment

In addition, it is not said explicitly either if the "amount of variance" in line 91 refers specifically to phenotypic, mutational, or genetic variance; it is thus unclear what exactly the model's prediction is. Is strength of selection expected to be proportional to phenotypic, mutational, or genetic variance? Are these variances also proportional to each other under this model? Clarification would be desirable.

This model of selection is essentially embeded in existing models of apparent stabalilzing selection, which we now clarify in the main text (line 126-128). But importantly, Jiang and Zhang develop essentially an identical model to describe apparent selection on wing shape in the very context of fly wings, where selection is described as total euclidean distance from the mean shape. We now cite this model and have added key discussion to the relevant parts of the main text (Line 128-132)

Line 98-99 "consistent with a model of apparent stabilizing selection against total multivariate distance from the mean wing shape (as proposed by ref12)."

Line 125-131 "Although this concept has been developed14 in the context of apparent stabilizing selection on phenotypic scores on gmax, the same reasoning extends beyond the first principal component of variation. Indeed, Jiang and Zhang12 developed precisely such a simulation model of apparent selection via pleiotropy on total Euclidean distance from the mean in order to explain stasis in fly wing shape; our estimate of selection provides direct empirical support for their model."

3. Connection to previous analyses of fly wing traits
In an introductory paragraph, the authors mentioned results of Houle et al. (2017). However, the reported similarity between the M matrix and G or R matrices, which is mentioned in line 71-74, is exaggerated due to their biased method for matrix comparison; nevertheless, there is still significant alignment between them (Jiang and Zhang, 2020). I suggest this issue being mentioned as long as this very result of Houle et al. (2017)'s is being mentioned. Houle et al. later proposed an alternative way of matrix comparison in a preprint, but in the same

manuscript they also acknowledged that their original method is not informative (Houle et al., 2020). The observation of very low evolutionary divergence relative to neutral expectation, which appears to be a motivator of this study, is an artifact like the scaling relationship, so it may be desirable to also clarify that the present study is “safe”.

We now cite this paper at the suggested point in the manuscript to clarify that there is some controversy over that result. We have kept further elaboration to a minimum here for reasons of space, since as the reviewer notes that potential artifact in the Houle et al. paper doesn’t affect our conclusions or motivations.

The scaling relationship between mutational variances and evolutionary rates of wing shape traits reported in Jiang and Zhang (2020), which was interpreted as that phenotypic dimensions with more mutational variance were under stronger (apparent) stabilizing selection, also appear consistent with the finding of this study. For this connection, I suggest that the previous result is mentioned, maybe in the sentence of line 112-117.

The reanalysis mentioned above (Jiang and Zhang, 2020) proposed an explanation for fly wing evolution that is essentially apparent stabilizing selection. They demonstrated using simulations that such a model can reproduce observed patterns, but could not directly test if the same mechanism did contribute to fly wing evolution (which is made possible by new data). Thus, I suggest that it is briefly mentioned, perhaps near the sentence of line 105-106.

We address both of the comments above here since they are related. We thank the reviewer for pointing us to the Jiang and Zhang reference, which we were unaware of. It is highly relevant, and also justifies our modelling approach, since they essentially develop the same fitness model to describle apparent stabalizing selection via pleotropy, but as the reviewer notes did not estimate selection but instead performed simulations. We now cite and discuss this paper at several points in our revised paper. (line 74, 99)

Minor comments

Line 77-79: While the cited paper did use the word “evolvability” to refer to additive genetic variance (though with phenotypic variance used as a proxy), this is not a practice I would recommend, personally. I consider evolvability as a property that is affected by many factors including genetic and phenotypic variances and thus not a synonym of the latter. Thus, I would recommend using “additive genetic variance” or “phenotypic variance” here instead.

Fair point. We have changed “evolvability” to “variance” here, which can incompass Va or Vp.

Line 152-154: Cheverud (1984) would be relevant to cite here, as it was an early theory paper that connected variation to developmental constraints.

added

Line 166-167: I agree that, in general, we cannot predict the movement of adaptive peaks very well due to the lack of first principles. There has been some hypothesis about this though, and the idea of a “selection line of least resistance” may be relevant to cite here (Arnold, 2023; the idea can be seen in his earlier papers, but I’m only citing his recent book here for convenience).

Yes, we aware of Arnold’s idea here. We cite it now in this place, referring to his book. However, we do not expand on this idea for two reasons. First, the pragmatic reason of space. Second, I am not totally convinced that this idea- which is that adaptive peaks tend to move/wobble in the direction characterized by weaker selection – is really based on first principles, or anything other than intuition. Schluter makes this very criticism of Arnold’s idea in the paper we cite at this point in our manuscript. This is certainly an interesting topic, but regardless the disagreement on this point between two of the most prominent thinkers in the field makes us hesitant to say more given space limations. At least, it is clear there is no consensus for how we should expect optima to move on a macroevolutionary landscape.

I find it a bit difficult to read Fig. 2A with four sets of data points put together and three of them (G, M, R) in somewhat transparent color. I would prefer them being plotted separately and placed side by side.

We appreciate this concern, but rather than expand this one panel, we have added another panel (2B) with brighter colors and which hopefully illustrates the general pattern more clearly. This is in response to the reviewers other concern above about whether selection is proportional to phenotypic variance. In the process of modifying figure 2 with this new panel, we have shrunk 2A, which we realize is opposite of what the reviewer is suggesting here. We did this because we think panels 2B and 2C are more illustrative of the key results. We are happy to present these panels differntly if the reviewers and/or editor feel strongly.

I think it is better to note that “glmm” means “generalized linear mixed model” when it appears for the first time (line 96), in case readers get confused. I also suggest writing abbreviations like GLMM in capital letters for better readability.

Corrected

Note that I did not take format requirements that might exist into account when making suggestions. Apologies for any inconvenience this might cause.

Our manuscript is now slightly longer, but we believe our arguments are strengthed substantially by these additions

References

Cheverud J. M. (1984) Quantitative genetics and developmental constraints on evolution by selection. J. Theor. Biol.
Jiang D. and Zhang J. (2020) Fly wing evolution explained by a neutral model with mutational pleiotropy. Evolution.
Houle D., Bolstad G. H., Hansen, T. F. (2020) Fly wing evolutionary rate is a near-isometric function of mutational variation. BioRxiv.
Arnold S. (2023) Evolutionary Quantitative Genetics. (book)

Reviewer #1:
Remarks to the Author:
I would like to thank the Authors for their careful revisions to the manuscript. My previous concerns regarding size and allometry have been fully addressed through the additional analyses and responses provided.

I do, however, find the discussion of effective population size (Ne) somewhat unclear (or incomplete). In particular, I think it's important to note in the main text that some published estimates exceed one million. The current version simply states the estimates in melanogaster are "very large". I suspect that most readers will find the numbers presented in the manuscript to be small, and being upfront about the range of estimates will make this more transparent. I don't disagree with the Authors' conclusions, but providing a more explicit and balanced range of estimates seems appropriate.

We appreciate this and have revised this section to clarify and be more explicit. It now reads "We find that our estimate of selection readily explains the constrained divergence in wing shape across the clade; the value of N_e necessary to produce an expected stationary variance consistent with the observed[11] among-species phenotypic variance, given our estimate of selection, is low (N_e = 140, with an upper confidence limit approaching 5000) but consistent with non-model wild *Drosophila* (e.g. harmonic mean of estimates from *D. buzzati* = 174 [22]) as well as *melanogaster* evolve-and-resequence estimates (harmonic mean = 206 [23]), although inconsistent with some estimates of melanogaster N_e exceeding 1 million[24]. "

I have no further comments or suggestions beyond this point, aside from recommending a check of the spelling of "Ornstein–Uhlenbeck" (line 143).

Corrected

Reviewer #2:
Remarks to the Author:
I greatly appreciate what the authors have done to address my questions. I think this version of the manuscript is overall strong and well written. In particular, the updated Figure 2 is a lot more informative now. I only have some minor comments for this version.

1. While Fig. 2A is shrunk, I agree that the newly added Fig. 2B is helpful and more than made up for it. It is now shown that the four regression slopes are not significantly different from -1. I think it would also be worth it to mention in the main text that the four CIs also overlap, which would strengthen the statement that the four slopes are "similar" to each other. Hopefully this only adds one more sentence.

Added. "for all 4 sets of phenotypic scores, sign-log-log regression of estimated selection differentials on the phenotypic standard deviation yields slopes that are significantly negative (all $P < 0.001$) with confidence intervals overlapping –1 and each other estimate (Figure 1B)."

Note that the figure in the PDF file I can access now has much lower resolution than the original version's PDF; the figure in the marked Word document is fine.

We have uploaded vector PDFs for all figures

2. In the main text, where X-axes of Fig 2A-B are referred to simply as "phenotypic score on [P, M, G, or R matrix]" (line 89). It is not entirely clear, given the main text alone, how these scores were obtained and how they differed from the "original" scores. Thus, it might be better to say "phenotypic score on principal components [of P, M, G, or R matrix]" instead (as in Methods, line 276), which I think is clearer and would not add too much more text. Alternatively, the result can be described as "relationship between selection differential and mutational/genetic/evolutionary variance", as in line 86.

Revised. "While the pattern is strongest when selection is estimated on phenotypic scores on the principal components of the phenotypic covariance matrix **P,** we find a similar pattern when selection is inferred on phenotypic scores on principal components of an existing[11] estimate of the genetic covariance matrix **G**, the mutational covariance matrix **M**, and the macroevolutionary rate matrix"

I should have brought this up in the last round of review since this is not a new issue that arose in the revision.

Apologies for any inconvenience.

3. Line 275: It is added that it is the "heterozygous" M-matrix (the matrix denoted as M_het in Houle et al., 2017, supposedly?) that was used. I appreciate the clarification, but also wonder why they would like to focus on M_het instead of M_hom (e.g., Houle et al. put more emphasis on M_hom and presented it as a "main" result).

Clarified. These estimates are similar and conclusions remain the same regardless of which is used. "We projected eigenvectors of **P**, as well as previous estimates (from [11]) of **G**, heterozygous **M** (conclusions the same for homozygous estimate), and **R**, on the centered shape data to obtain phenotypic scores on the principal components of these matrices"
